# A novel method for early detection of colorectal cancer based on detection of methylation of two fragments of syndecan-2 (SDC2) in stool DNA

**DOI:** 10.1186/s12876-022-02264-3

**Published:** 2022-04-18

**Authors:** Liang Ma, Geng Qin, Fei Gai, Yongwei Jiang, Zhan Huang, Hui Yang, Shukun Yao, Shiyu Du, Yongtong Cao

**Affiliations:** 1 Clinical Laboratory, China-Japan Friendship Hospital, Beijing, 100029 China; 2 Department of Gastroenterology, China-Japan Friendship Hospital, Beijing, 100029 China; 3 Medical Business Unit, Amoy Diagnostics Co. Ltd., Xiamen, 361026 China

**Keywords:** Colorectal cancer, Combined detection, Methylation, *SDC2*, Stool DNA

## Abstract

**Background:**

Methylated *SDC2* has been proved as a diagnostic marker for human colorectal cancer (CRC), noninvasive stool DNA-based methylation testing also emerges as a novel approach for detecting CRC. The aim of this study was to evaluate the clinical performance of stool DNA-based *SDC2* methylation test by a new qPCR detection reagent for early detection of CRC.

**Methods:**

A new qPCR detection reagent contained two differentially methylated regions in SDC2 CpG islands for the detection of CRC was used in this study. Performance of the SDC2 methylation detection reagent was evaluated by analyzing limit of detection, precision, and specificity. The effect of interfering substances on assay performance was also tested. 339 subjects (102 CRC patients, 50 patients with advanced adenomas, 39 patients with non-advanced adenomas, 18 colitis patients and 130 normal individuals) from the China-Japan Friendship Hospital were evaluated. Approximately 2.5 g of stool sample was collected from each participant. Stool DNA was extracted and bisulfite-converted, followed by qPCR assay, which contained two pairs of primers for the methylation detection of two fragments of the SDC2 gene (named SDC2-A and SDC2-B). The diagnostic value of this test in CRC was evaluated by calculating receiver operating characteristic (ROC) curve, and value of the area under the curve (AUC).

**Results:**

The test kit was able to detect methylated *SDC2* in stool DNA samples with concentrations as low as 90 copies/μL in 100% of replicates. The sensitivity for detecting CRC by methylated SDC2-A alone was 85.29% (95% CI 77.03–91.00%) with a specificity of 96.15% (95% CI 91.08–98.58%). The sensitivity by methylated SDC2-B alone was 83.33% (95% CI 74.82–89.42%) with a specificity of 97.69% (95% CI 93.14–99.51%). However, when methylated SDC2-A and methylated SDC2-B were combined, the sensitivity for CRC detection improved to 87.25% (95% CI 79.27–92.53%) with a specificity of 94.62% (95% CI 89.11–97.56%). Further, the detection reagent achieved ROC-AUC 0.874 (95% CI 0.822–0.927) for SDC2-A, 0.906 (95% CI 0.859–0.952) for SDC2-B, and 0.939 (95% CI 0.902–0.977) for SDC2-Combine A&B.

**Conclusions:**

This study validated the capability of stool DNA-based *SDC2* methylation test for early screening of CRC, and combined detection of two fragments of *SDC2* gene could improve detection sensitivity.

**Supplementary Information:**

The online version contains supplementary material available at 10.1186/s12876-022-02264-3.

## Background

Colorectal cancer (CRC) is the most common malignancy, ranking the third in incidence and the second in mortality, all over the world [1]. China indicated lower rates of incidence with 517,000 new cases (14.2 per 100,000), mortality with more than 245,000 deaths (7.4 per 100,000), than most developed countries. However, China had a higher mortality/incidence ratio (52.1%) and lower 5-year survival rate [2–4], due to more than 85% of CRC was found to be advanced. Even after comprehensive treatment such as surgery, radiotherapy and chemotherapy, targeted therapy, 5-year survival rate was still significantly lower than 40% in China. Therefore, early CRC screening program is urgently promoted in China [5].

Similar to most countries, a two-step screening strategy has been recommended in China for population screening, a quantitative high-risk factor questionnaire and fecal occult blood test (FOBT) as the primary screening, with a full colonoscopy for follow-up [6, 7]. At present, colonoscopy is accurate for the diagnosis of CRC [8]. However, due to its invasiveness, dietary restriction requirements, extensive bowel preparation, poor doctor–patient communication, and no insurance coverage, its compliance rate is still very low (~ 20%) in China [9]. Guaiac fecal occult blood test (gFOBT) and fecal immunochemical test (FIT) are the two commonly use method for FOBT. However, gFOBT has low sensitivity in detecting CRC and its precancerous lesions [10], which cannot significantly reduce the incidence of CRC. In addition, the test results are easily interfered by food, drugs and other factors, reaching a relatively high false positive rate. The key disadvantage of FIT is the low sensitivity of detecting advanced adenomas (As defined by the US Multi-Society Task Force on Colorectal Cancer [11], advanced neoplasia is defined as an adenoma with size ≥ 10 mm, villous histology, or high-grade dysplasia. While, if none of the above features were present, it will define as non-advanced adenomas), which is less than 50% even in high-risk populations [12, 13].

Stool DNA aberrant detection from colorectal exfoliated cells, including gene mutation and/or methylation has become the potential screening method in recent years. Especially the aberrant methylation detection, which is chemically and biologically stable, is readily detectable in blood and stool [14, 15]. The specific gene including *SEPT9* [16, 17], *SDC2* [18, 19], *SFRP2* [18, 20], and *TFPI2* [21] have found to associate with CRC and precancerous lesions. In previous studies, the performance of commercial kits (Epi proColon® 2.0 assay and ColoVantage®) based on plasma methylated *SEPT9* screening is associated with CRC stage, with low sensitivities in early-stage CRC and advanced adenomas [22]. While, Cologuard, the first stool-based CRC screening test approved by the US Food and Drug Administration (FDA) includes two methylated DNA biomarkers, *BMP3* and *NDRG4*. It could detect 92.3% of CRC and 42.4% of advanced adenomas with a specificity of 86.7% [13]. Stool samples can be collected at home, and it is very suitable for those who worry about privacy or are too afraid of cross-infection (such as Covid-19) in hospital. However, its feasibility for early detection of CRC and precancerous lesions in the Chinese population remains inconclusive.

*SDC2* belongs to syndecan family and encodes an integral membrane protein that is heavily glycosylated [23]. SDC2 protein acts as a receptor for extracellular matrix components, and it has been reported to play a critical role either as a tumor suppressor, such as in osteosarcoma [24], or an oncogene, promoting survival and metastases in breast cancer [25]. Hypermethylation of *SDC2* promoter region is a frequent epigenetic change occurs during the development of colorectal neoplasms. As described by Oh T et al. in 2013, methylation target regions of SDC2 gene exhibited a significantly higher methylation level in primary tumors (100%, 12/12), compared with paired, adjacent nontumor tissue (*P* ≤ 0.0011) [26]. As described by Bartak et al. in 2017, DNA methylation of SDC2 was observed in 89.4% (42/47) in the plasma fraction of patients with CRC, and 81.1% (30/37) of adenoma patients. Nevertheless, this marker was found to be methylated in only 2.7% (1/37) of healthy control samples [18] and has been successfully detected in a variety of clinical specimens including tissue [27], blood [26], and stool [19, 28] samples. Thus, in this study, we evaluated the feasibility of methylated *SDC2* as a biomarker for early CRC detection in stool specimens in the Chinese population. Since, in present, most stool DNA-based methylation assays used *SDC2* single gene or *SDC2* combined with *SFRP2* or other genes as the biomarker for CRC screening [29–31]. Compared with *SDC2* single gene test, *SDC2* combined with other gene test has a higher sensitivity, but the cost was also higher than that of single gene test. Therefore, we adopted the method of internal combination of *SDC2* gene (detecting two different methylation regions simultaneously), which could not only improve the accuracy of detection, but also save the cost of detection.

## Methods

### Analytical performance of *SDC2* methylation detection reagent

To determine the sensitivity of detection for methylated *SDC2* DNA, colorectal cancer cell line HCT116 diluted with negative culture medium was prepared as reference sample, and positive cell line samples with concentration gradient of 120 copies/μL, 110 copies/μL, 100 copies/μL, 90 copies/μL, and 80 copies/μL were tested using this *SDC2* methylation detection reagent, each concentration sample was repeated for 20 times.

Assay specificity of the detection reagent was assessed by testing *SDC2* methylation in constructed plasmids containing methylated-CpG islands sequence of other colorectal cancer-related genes, including *Septin9*, *BMP3* and *NDRG4*.

Three batches of reagents were used to test *SDC2* gene methylation negative, weakly positive (2 times as strong as the detection limit) and strongly positive (80 times as strong as the detection limit) reference samples for 20 times in consecutive 3 days, respectively. % of coefficient of variance (CV) of the obtained Ct values was calculated to evaluate the intra-batch/inter-batch detection precision of the detection reagent.

Following the Clinical Laboratory Standards Institute (CLSI) guideline about interference testing, EP7-A2 [32], and Guide to interference testing in clinical chemistry, WS/T 416-2013 [33], the effect of interfering substances on assay performance was evaluated using methylated *SDC2*-positive and negative stool samples spiked with 24 potential interfering substances selected based on clinical applications and diet habits in China (Additional file 1: Table S1).

### Sample collection

339 participants who underwent colonoscopy at China-Japan Friendship Hospital from July 2019 to November 2019 were enrolled in the study. All colonoscopies were performed by board-certified endoscopists. Based on results of complete colonoscopy and histopathology outcome, subjects were categorized as follows: CRC (Guidelines for the Diagnosis and Treatment of Colorectal Cancer in China, 2019 Edition), advanced adenoma (size greater than or equal to 1.0 cm, greater than 25% villous component, or high-grade dysplasia), non-advanced adenoma (size less than 1.0 cm in the greatest dimension), colitis (colonoscopy showed mucosal hyperemia and edema, but the pathological diagnosis ruled out ulcerative colitis and Crohn's disease), and no evidence of disease (negative results on colonoscopy, normal control). Stool samples (> 2.5 g) were collected at least 1 day before bowel preparation for colonoscopy, deposited into a storage tube prefilled with preservative buffer (Amoy Diagnostics Co. Ltd., Xiamen, China) for immediately methylation detection of human *SDC2* gene, or stored at − 20 ± 5 ℃ for no more than 1 month. The study was blind. In order to eliminate the influence of known colonoscopy results, before the experimental operation, EXCEL was used to generate random numbers, and used random numbers to number and identify the collected stool samples, replacing the original traceable sample numbers. The blinding work was carried out by independent blinding personnel, and blinding would be uncovered after the interpretation of test results was completed. This study was approved by the Institutional Review Board of the China-Japan Friendship Hospital (No. No. 2019-50-Q07), and the informed consent was obtained from all participating patients and healthy control subjects.

### DNA isolation and bisulfite treatment

Stool DNA extraction and bisulfite conversion and purification were performed with a commercial extraction and bisulfite conversion kit (Fecal BisDNA, Amoy Diagnostics Co. Ltd., China) according to the manufacturer’s instruction. The extracted and transformed stool DNA was tested immediately. Otherwise, the transformed stool DNA was stored at − 20 ± 5℃, and the test was completed within 1 month.

### Methylation detection of human *SDC2* gene

Real-time PCR amplification was performed on SLAN-96S Real-Time PCR System (Shanghai Hongshi Medical Technology Co., Ltd., China) to detect the methylated *SDC2* gene. And methylation of two different fragments (named SDC2-A and SDC2-B) of *SDC2* gene was detected (Amoy Diagnostics Co. Ltd., Xiamen, China). ACTB was used as an internal control. PCR reactions for the two methylation fragments of *SDC2* and *ACTB* were run in a single tube simultaneously. PCR amplification was performed in a reaction volume of 40.0 μL containing 5–15 ng of sample DNA (5.0 μL), 0.3 μL of *SDC2* mixed enzyme (DNA polymerase, uracil-N-glycosylase, Amoy Diagnostics Co. Ltd., Xiamen, China), and the reaction mix was brought to the final volume with SDC2-A reaction liquid or SDC2-B reaction liquid (Amoy Diagnostics Co. Ltd., Xiamen, China). Thermal cycling conditions were as follows: 95 °C for 7 min; 15 cycles at 95 °C for 25 s, 60 °C for 20 s and 72 °C for 20 s; 30 cycles at 93 °C for 25 s, 56 °C for 35 s and 72 °C for 20 s. The interpretation criteria of test results were shown in Additional file 1: Table S2. The sequences of primers and probes were: ACTB, Forward: 5ʹ- CAC CAA CCT CAT AAC CTT ATC -3ʹ and Reverse: 5ʹ- TAA TAC CTA CAC CCA CAA CAC -3′ and probe: TTT GTT TTT TTG ATT AGG TGT TTA AGA; fragment SDC2-A, Forward: 5′- TAA TTT CGT GTC GGG AGT GTA GAA ATT -3′ and Reverse: 5′- AAG CGA GCG TTT TCG AGT TTC GAG T -3′ and probe: TAA GTG AGA GGG CGT CGC GTT TTC G; fragment SDC2-B, Forward: 5′- CAC GCA AAC CAC CAA ACC CAA AAT A -3′ and Reverse: 5′- CTC GTA ACT TCA AAC ACC CTA AAC GA -3′ and probe: CGC CTA ACC CAC TCA CCG ACT CCG.

### Statistical analysis

Since the methylation test was performed on two different fragments of *SDC2* gene in the study, two test results were generated for each sample, so there would be multiple results interpretation methods. Here, the methylation analysis result was defined as the Δ threshold cycle (ΔCt) value (ΔCt = number of copies of methylated DNA-the number of copies of ACTB), and any positive result from fragment A or B will be considered as a positive test result. The diagnostic performance was evaluated in terms of the sensitivity, specificity, and area under the receiver operator characteristic (ROC) curve (AUC). Chi-square test was performed to evaluate the correlation of diagnosis results with clinical characteristics. Statistical calculations were performed using SPSS (version 19.0). *P* value < 0.05 was considered as statistically significant.

## Results

### Performance evaluation of *SDC2* methylation detection reagent

To determine the limit of detection (LOD) for methylated *SDC2* DNA, the detection concentration gradient was set as 120 copies/μL, 110 copies/μL, 100 copies/μL, 90 copies/μL and 80 copies/μL. The two fragments of SDC2 in the detection reagent were tested repeatedly for 20 times, and the detection limit was considered when the positive detection was more than 19 times. The test kit was able to detect methylated *SDC2* in stool DNA samples with concentrations as low as 90 copies/μL in 100% of replicates (Table 1). Precision evaluation results of *SDC2* methylation test were described in Table 2. It was considered acceptable if CV was less than 5%. The three type reference samples were repeatedly tested 20 times with the detection reagent, and the CV statistics were conducted. The test showed acceptable repeatability, with CV ranging from 1.09 to 3.18% and 0.67 to 2.29% for SDC2-A and SDC2-B, respectively. The methylation test results of *Septin9*, *BMP3* and *NDRG4* genes using this detection reagent were all "No Ct". Interfering substances tested in this study had no effect on test performance of the detection reagent.

**Table 1:** Detection limits of the *SDC2* methylation detection reagent

Detection target	120 copies-Ct value (%)		110 copies-Ct value (%)		100copies-Ct value (%)		90 copies-Ct value (%)		80 copies-Ct value (%)	
	SDC2-A	SDC2-B	SDC2-A	SDC2-B	SDC2-A	SDC2-B	SDC2-A	SDC2-B	SDC2-A	SDC2-B
Methylation	100	100	100	100	100	100	100	100	90	100
Internal control	100	100	100	100	100	100	100	100	100	100

**Table 2:** Precision evaluation results of the *SDC2* methylation detection reagent

Detection target	Negative sample (%)		Weakly positive sample (%)		Strongly positive sample (%)	
	SDC2-A	SDC2-B	SDC2-A	SDC2-B	SDC2-A	SDC2-B
Methylation	/	/	3.18	2.29	1.09	0.67
Internal control	0.79	0.62	1.16	0.54	1.30	0.61

### Clinical characteristics of participants

**Fig. 1:**
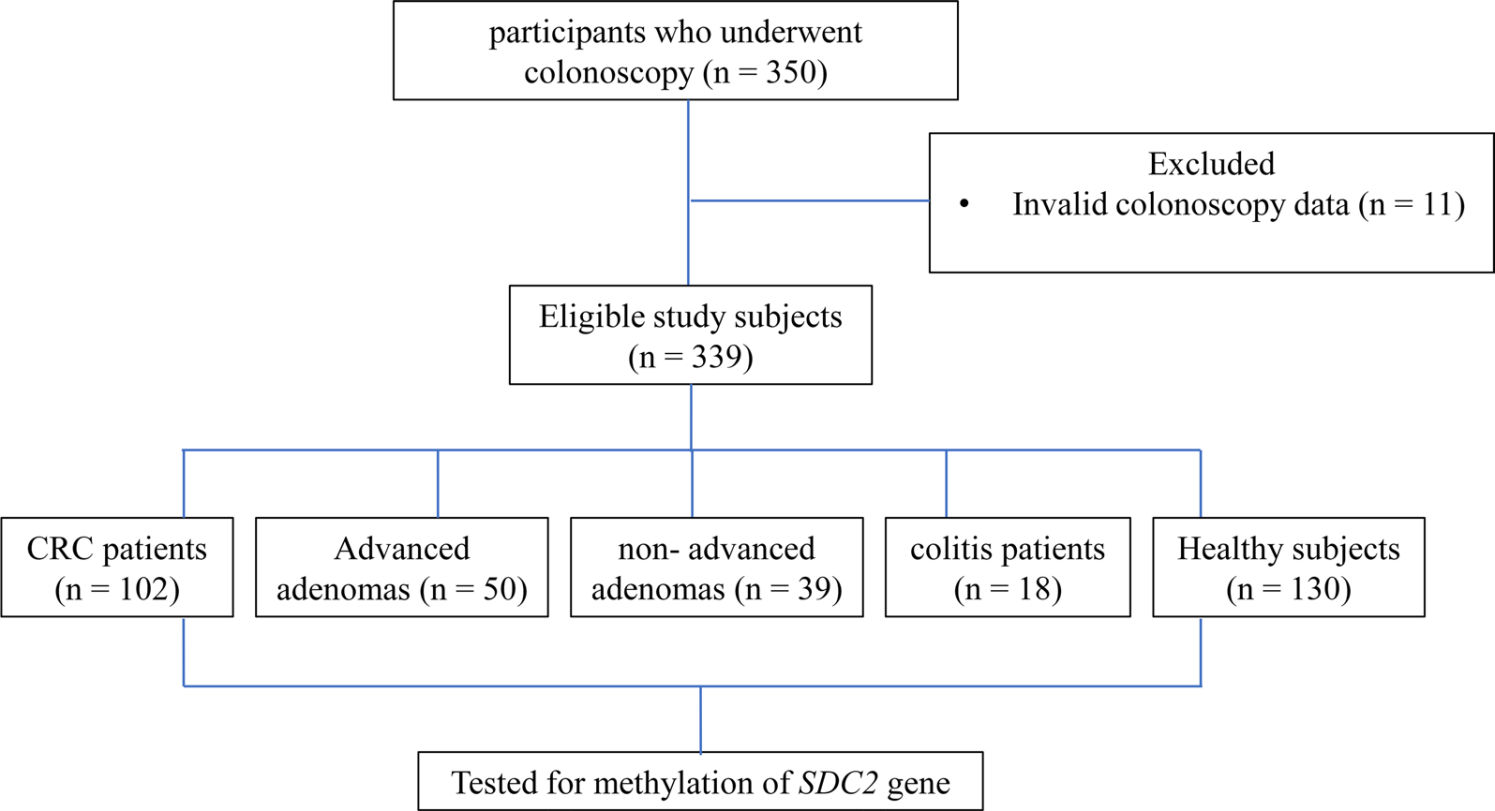
Flowchart of disposition of the study participants

The median age of all 339 participants were 57 years (range, 20 to 89 years), 173 (51.03%) were male, and 166 were female (48.97%). According to clinical diagnosis, the participants were assigned into five categories, including, 102 CRC patients, 50 patients with advanced adenomas, 39 patients with non- advanced adenomas, 18 colitis patients and 130 individuals with negative colonoscopy (normal control), the flowchart of disposition of the study participants is shown in Fig. 1.

Of all CRC patients, 49.02% were male. Most of the tumors were at stage II and III (60.78%). Of normal controls, 45.38% were male individuals. The clinical characteristics of the participants were shown in Table 3.

**Table 3:** Demographics of study subjects (N = 339)

Characteristics	Overall	CRC n (%)	Advanced adenomas n (%)	Non‐advanced adenoma n (%)	Colitis n (%)	Normal control n (%)	*P* value
Number	339	102	50	39	18	130	
*Gender*							0.054
Male	173 (51.03%)	50 (49.02%)	35 (70.00%)	19 (48.72%)	10 (55.56%)	59 (45.38%)	
Female	166 (48.97%)	52 (50.98%)	15 (30.00%)	20 (51.28%)	8 (44.44%)	71 (54.62%)	
*Median age (years, range)*							< 0.001
< 35	40 (11.81%)	3 (2.94%)	2 (4.00%)	4 (10.26%)	5 (27.78%)	26 (20.00%)	
35–45	47 (13.86%)	10 (9.80%)	1 (2.00%)	1 (2.56%)	4 (22.22%)	31 (23.85%)	
46–55	65 (19.17%)	13 (12.75%)	12 (24.00%)	13 (33.33%)	4 (22.22%)	23 (17.69%)	
> 55	187 (55.16%)	76 (74.51%)	35 (70.00%)	21 (53.85%)	5 (27.78%)	50 (38.46%)	
*Clinical stage*							/
0	/	4 (3.92%)	/	/	/	/	
I	/	11 (10.78%)	/	/	/	/	
II	/	28 (27.45%)	/	/	/	/	
III	/	34 (33.34%)	/	/	/	/	
IV	/	2 (1.96%)	/	/	/	/	
Not determined	/	23 (22.55%)	/	/	/	/	

### Clinical performance of the *SDC2* methylation detection reagent for CRC screening in stool DNA

**Fig. 2:**
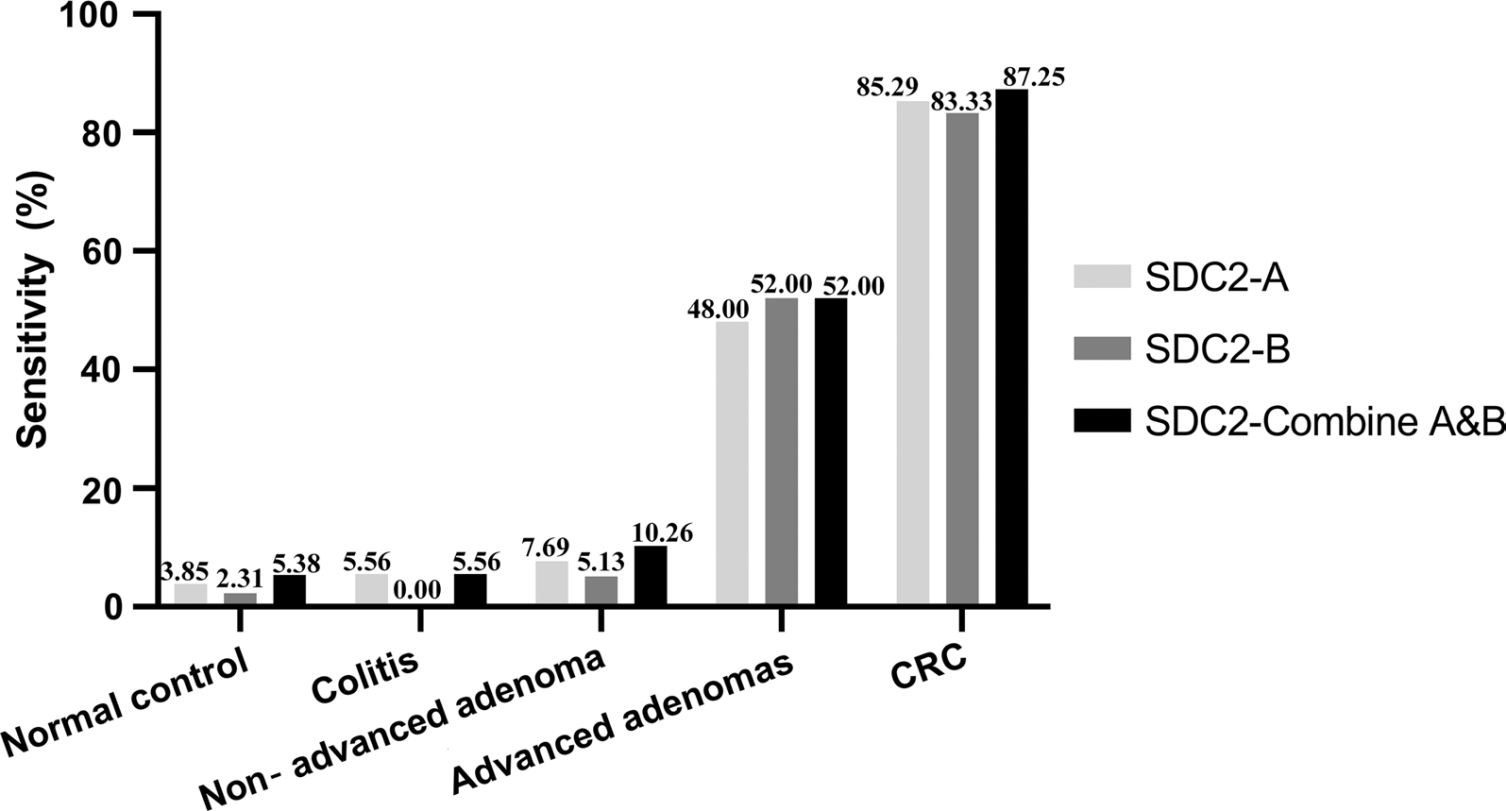
Detection rate of methylated *SDC2* in CRC patients, advanced adenomas patients, non-advanced adenomas patients, colitis patients and normal control

339 valid samples obtained from the participants were tested using the *SDC2* methylation detection reagent, the result showed higher frequency of aberrant *SDC2* methylation in CRC patients than that in normal controls (Fig. 2).

**Fig. 3:**
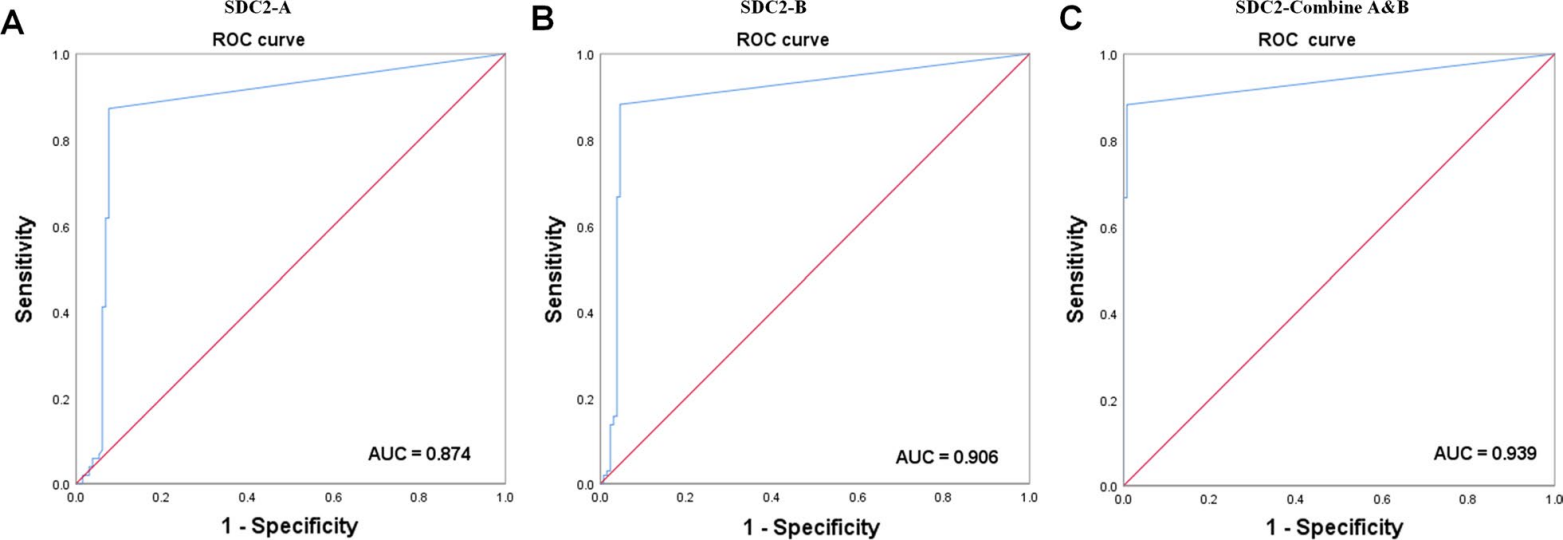
ROC curve was plotted for CRC patients vs. normal control, AUC was indicated. **A** ROC curve for methylated SDC2-A. **B** ROC curve for methylated SDC2-B. **C** ROC curve for SDC2-Combine A&B

Sensitivity and specificity of *SDC2* methylation in stool DNA samples from all participants are summarized in Table 4. Although there was no statistical difference, SDC2-Combine A&B for CRC had a higher sensitivity of 87.28% (89/102, 95% CI 85.8–93.6%), while the sensitivity of SDC2-A and SDC2-B for CRC were 85.29% (87/102, 95% CI 77.03–91.00%) and 83.33% (85/102, 95% CI 74.82–89.42%), respectively. For 130 subjects with totally negative results on colonoscopy, the specificity of SDC2-Combine A&B, SDC2-A, and SDC2-B was 94.62% (123/130, 95% CI 89.11–97.56%), 96.15% (125/130, 95% CI 91.08–98.58%), and 97.69% (127/130, 95% CI 93.14–99.51%), respectively. ROC curves of methylated SDC2 for CRC detection were shown in Fig. 3. AUC for methylated *SDC2* tested by SDC2-A and SDC2-B were 0.874 (95% CI 0.822–0.927) and 0.906 (95% CI 0.859–0.952), respectively. In contrast, SDC2-Combine A&B improved AUC to 0.939 (95% CI 0.902–0.977). Sensitivities for individual characteristics of CRC patients were analyzed and compared among SDC2-Combine A&B, SDC2-A, and SDC2-B. For each characteristic, detection sensitivity of SDC2-Combine A&B was higher than SDC2-A and SDC2-B, respectively, however, the difference was not significant (Table 5, *P* > 0.05).

**Table 4:** Sensitivity and specificity of the stool DNA test targeting methylated *SDC2*

	SDC2-Combine A&B	SDC2-A	SDC2-B	*P* value
	Sensitivity (95% CI)	Sensitivity (95% CI)	Sensitivity (95% CI)	
CRC	87.25% (79.27–92.53%)	85.29% (77.03–91.00%)	83.33% (74.82–89.42%)	0.732
Advanced adenomas	52.00% (38.51–65.20%)	48.00% (34.80–61.49%)	52.00% (38.51–65.20%)	0.899

	SDC2-Combine A&B	SDC2-A	SDC2-B	
	Specificity (95% CI)	Specificity (95% CI)	Specificity (95% CI)	
Normal control	94.62% (89.11–97.56%)	96.15% (91.08–98.58%)	97.69% (93.14–99.51%)	0.435
Colitis	94.44% (72.35–99.99%)	94.44% (72.35–99.99%)	100.00% (79.33–100%)	0.595
Non‐advanced adenoma	89.74% (75.85–96.51%)	92.31% (78.97–98.06%)	94.87% (82.21–99.48%)	0.697

**Table 5:** Sensitivity of the stool DNA test targeting methylated *SDC2* in different characteristics of CRC patients

	SDC2-Combine A&B Sensitivity (95% CI)	SDC2-A Sensitivity (95% CI)	SDC2-B Sensitivity (95% CI)	*P* value
*Gender*				
Male	86.00% (73.50–93.36%)	82.00% (68.98–90.46%)	84.00% (71.22–91.93%)	0.862
Female	88.46% (76.66–94.97%)	84.62% (72.21–92.26%)	86.54% (74.42–93.63%)	0.848
*Age*				
< 35	100.00% (38.25–100.00%)	100.00% (38.25–100.00%)	100.00% (38.25–100.00%)	/
35–45	70.00% (39.23–89.67%)	70.00% (39.23–89.67%)	70.00% (39.23–89.67%)	1.000
46–55	92.31% (64.58–99.99%)	84.62% (56.54–96.90%)	76.92% (49.06–92.50%)	0.554
> 55	88.16% (78.78–93.86%)	84.21% (74.24–90.89%)	88.16% (78.78–93.86%)	0.708
*Clinical stage*				
0	50.00% (15.00–85.00%)	50.00% (15.00–85.00%)	50.00% (15.00–85.00%)	1.000
I	90.91% (60.09–99.99%)	90.91% (60.09–99.99%)	90.91% (60.09–99.99%)	1.000
II	85.71% (67.89–94.92%)	85.71% (67.89–94.92%)	82.14% (63.94–92.59%)	0.913
III	88.24% (72.78–95.93%)	82.35% (66.11–92.03%)	88.24% (72.78–95.93%)	0.718
IV	100.00% (29.02–100.00%)	100.00% (29.02–100.00%)	100.00% (29.02–100.00%)	/
Not determined	91.30% (72.03–98.75%)	82.61% (62.26–93.63%)	86.96% (67.03–96.31%)	0.682

## Discussion

Stool DNA-based molecular marker tests have recently been proposed as a new option for screening early CRC. Methylated *SDC2* as a stool-based biomarker for CRC was noticed in recent years. Several recent studies reported that the sensitivity of methylated *SDC2* for all stage CRC screening with stool samples were 77.0–93.9% with specificity of 88.2– 98.1% [9, 28–31, 34–36] (Additional file 1: Table S3), respectively. The sensitivities of methylated *SDC2* from these studies were improved by calculation of the percentage of methylated *SDC2* with *ACTB* as the reference gene or using methylation-specific PCR (MSP). In this study, we introduced a new stool DNA-based early CRC screening assay, which combined two methylation fragments, SDC2-A and SDC2-B, in a single PCR reaction. Any positive result from fragment A or B will be considered as a positive test result, thus detection rate will be improved by joint detection and combined analysis. LOD of the present method was as low as 90 copies/μL in 100% of replicates, which was equal to the published pyrosequencing [37] and LTE-qMSP [28]. In addition, its high stability and excellent anti-interference ability also showed its clinical application value. In this study, detailed analytical performance for the *SDC2* methylation detection reagent were first provided. Then the performance of the test reagent was evaluated using stool DNA from clinical patients.

Several previous studies have examined the performance of DNA methylation biomarkers in stool DNA for early detection of CRC or precancerous lesions. To detect CRC in stool DNA, the sensitivity of HIC1 and vimentin genes was 42% and 46%, respectively, and the specificity was 100% and 90%, respectively, indicating a good specificity but unsatisfactory sensitivity [38, 39]. *SFRP2* showed a sensitivity of 77–90% with specificity of 77% for detection of CRC in stool samples, showing an excellent sensitivity but unsatisfactory specificity [40]. Recently, a sensitivity of 77.3–85.9% and specificity of 91.5–95% has been shown for the stool-based methylated *KCNQ* and *C9orf50* for all stage CRC detection. And when methylated *C9orf50* and *KCNQ5* were combined, the sensitivity for CRC detection was improved to 88.4% [41]. In addition, the methylation of multiple genes (*vimentin*, *NDRG4*, *BMP3*, and *TFPI2*) combine with *KRAS* mutations in stool DNA were reported by Ahlquist et al., this test was able to detect CRC and precancerous adenoma with sensitivities of 85 and 54%, respectively, at a specificity of 90% [42]. However, the cost of combined detection is relatively high. Among the DNA methylation markers for CRC, previous studies have confirmed that the abnormal methylation of *SDC2* occurred in almost all CRC tissues regardless of stage and was observed also in biopsies of various precancerous lesions while not detected in normal intestinal mucosal tissues. And according to the severity of the lesion, the methylation level of *SDC2* in tissue samples tended to increase [26, 28]. Thus, *SDC2* methylation test in stool DNA was chosen in this study.

A new *SDC2* methylation detection reagent was designed and applied in the present study, it combined two methylation fragments, SDC2-A and SDC2-B, in a single PCR reaction. If at least one out of two *SDC2* fragments from a subject was positive, the test was considered as positive. Overall sensitivity of this detection reagent for CRC (0–IV) was 87.25% with a specificity of 94.62%. These observed clinical sensitivity and specificity results were comparable with the result reported by Han et al. [36] and higher than aforementioned studies [9, 31]. Since most other markers have not been reported for more detailed evaluation of the clinical performance of detecting different stages of CRC, the sensitivity of methylated *SDC2* for all stage CRC could be compared to those of methylated *C9orf50* and *KCNQ5* [41], which were very similar among all three methylation markers. Therefore, using detection of two *SDC2* methylation fragments can improve clinical performance for the early diagnosis of CRC, without losing specificity. It has been widely accepted that age is a high‐risk factor for genome DNA methylation, and many tumor suppressor genes have been reported to be age‐dependent hypermethylated genes. However, the methylation of *SDC2* did not show a strong correlation with age in our study and previous studies [29, 30, 34]. Our study and previous publications demonstrated that *SDC2* methylation is independent of patients’ clinical features, including sex, age, the location of the tumors and clinical stage. Although one CpG site (cg25070637) in the promoter of *SDC2* was reported to be significantly associated with age (43), this site did not overlap with SDC2-A and SDC2-B according to the genomic locations.

This study has several limitations. Small size of the patient, especially stage I and stage IV CRC patients, and control groups was insufficient to evaluate the diagnostic value of the detection reagent. And the mean age of control group was younger than CRC patients. Thus, increasing the number of enrolled patients and comparable controls need to be considered in future studies.

Further studies are needed to collect detailed clinicopathological information of the patients and to analysis the relationship between *SDC2* methylation and clinicopathological characteristic. Furthermore, prospective studies are needed for intensive evaluation of stool DNA-based *SDC2* methylation screening.

## Conclusions

This study demonstrated that this *SDC2* methylation detection reagent exhibited relatively high sensitivities and specificity for the detection of all stage CRCs using stool samples noninvasively. However, further large-scale studies are required to validate the clinical utility of this test in population-based CRC screening.

## Supplementary Information

**Additional file 1. Table S1**. Potentially interfering substances tested in this study.** Table S2**. The interpretation criteria of test results.** Table S3**. Stool DNA test for methylated SDC2 in different studies.

## Data Availability

The authors confirm that all data underlying the findings are fully available without restriction. All relevant data are within the paper and its supporting information files. The Excel file of test results of the research subjects has been uploaded to the Science Data Bank and can be downloaded via the following link: https://www.scidb.cn/s/feU3Ef..

## Abbreviations

ACTB: β-Actin
AUC: Area under the curve
CRC: Colorectal cancer
CV: Coefficient of variance
LOD: Limit of detection
ROC: Receiver operator characteristic curve
SDC2: Syndecan-2
ΔCt: Δ Threshold cycle value
