# Supplementary material for: A novel method for early detection of colorectal cancer based on detection of methylation of two fragments of syndecan-2 (SDC2) in stool DNA
Source: BMC Gastroenterol. 2022 Apr 18;22:191. doi: 10.1186/s12876-022-02264-3 (PMC9014784; doi:10.1186/s12876-022-02264-3)
Supplement: Supplementary file 1 — Additional file 1. Table S1. Potentially interfering substances tested in this study. Table S2. The interpretation criteria of test results. Table S3. Stool DNA test for methylated SDC2 in different studies. [file 12876_2022_2264_MOESM1_ESM.docx]

**Table S1.** Potentially interfering substances tested in this study

| Interfering substance types | Interfering substances |
| --- | --- |
| Antidiarrheal and laxative | Berberine hydrochloride  Tongbianling capsule  Mannitol |
| Common gastrointestinal drugs | Domperidone  Vitamin U,Belladonna and Aluminium Dispersible Tablets |
| Antibacterial drugs | Penicillin  Cefixime  Tetracycline  Levofloxacin |
| Antipyretic analgesic | Ibuprofen |
| Medicine for cold | Ganmaoling capsule |
| Antihemorrhoidal drug | Hemorrhoids cream |
| Antacids | Cimetidine  Omeprazole |
| Food | Vegetable oil  Animal oil  Alcohol  Vinegar |
| Endogenous components | Hemoglobin  Ferritin  Bilirubin  Albumin  Triglyceride |

**Table S2.** The interpretation criteria of test results

| Gene | Fluorescence channel | Positive test value | Interpretation of result |
| --- | --- | --- | --- |
| SDC2-A | FAM | 10.0 | ΔCt value<10.0 is positive, otherwise negative |
| SDC2-B | FAM | 10.5 | ΔCt value<10.5 is positive, otherwise negative |
| ACTB | VIC | 21 | Ct value≤ 21 is valid, otherwise invalid |

**Table S3.** Stool DNA test for methylated SDC2 in different studies

| Author (year)  PMID | Total subjects (N) | Colorectal cancer (N) | Healthy control (N) | Sensitivity (95% CI) | Specificity (95% CI) | Detection method |
| --- | --- | --- | --- | --- | --- | --- |
| Li et al. (2021)  PMID: 33955718 | 252 | 198 | 54 | 93.9% | 98.1% | Methylation-Specific PCR |
| Zhang et al. (2021)  PMID: 33958894 | 180 | 61 | 53 | 77.0% | 98.1% | Methylation-Specific PCR |
| Zhao et al. (2021)  PMID: 33854626 | 129 (validation cohort) | 58 | 38 | 86.2% | 97.4% | Quantitative real-time PCR |
| Su et al. (2021)  PMID: 33393623 | 138 | 62 | 76 | 77.4% (65.0–87.1%) | 88.2% (72.1–92.5%) | Quantitative real-time PCR |
| Zhao et al. (2020)  PMID: 32625237 | 104 (validation cohort) | 39 | 59 | 87.2% | 96.6% | Quantitative real-time PCR |
| Wang et al. (2020)  PMID: 33126908 | 1110 | 359 | 713 | 83.8% (79.5–87.4%) | 98.0% (96.6–98.9%) | Methylation-Specific PCR |
| Han et al. (2019)  PMID: 30876480 | 585 | 245 | 245 | 90.2% (85.8–93.6%) | 90.2% (85.8–93.6%) | Methylation-specific PCR coupled with linear target enrichment (LTE-qMSP) |
| Oh et al. (2017)  PMID: 29225717 | 93 | 50 | 22 | 90.0% (78.2–96.6%) | 90.9% (70.8–98.6%) | Methylation-specific PCR coupled with linear target enrichment (LTE-qMSP) |
| Niu et al. (2017)  PMID: 28619831 | 497 | 196 | 179 | 81.1% | 93.3% | Methylation-Specific PCR |
| Current study | 339 | 102 | 130 | 87.25% (79.27–92.53%) | 94.62% (89.11 – 97.56%) | Quantitative real-time PCR |
